# Patella dislocation: an online systematic video analysis of the mechanism of injury

**DOI:** 10.1186/s43019-020-00031-w

**Published:** 2020-05-27

**Authors:** V. Dewan, M. S. L. Webb, D. Prakash, A. Malik, S. Gella, C. Kipps

**Affiliations:** 1 Department Of Trauma & Orthopaedics, Royal Wolverhampton NHS Trust, Wolverhampton, UK; 2 University College London, London, UK; 3 St George’s Hospital, London, UK; 4 Sandwell & West Birmingham NHS Trust, Birmingham, UK

**Keywords:** Patellofemoral dislocation, Mechanism of injury, Systematic video analysis, Patella

## Abstract

**Background:**

The mechanism of injury (MoI) for a patellar dislocation has not been fully established. The aim of this study was to use systematic video analysis to determine the MoI of a patella dislocation.

**Methods:**

A systematic search was conducted of three video sharing websites and three popular search engines to identify videos demonstrating a patellar dislocation. Videos were reviewed by three surgeons trained in systematic video analysis, who commented on the position of the lower limb and the situation in which the injury occurred. The results were reviewed to build a consensus of the MoI for each video. Statistical analysis was conducted for interobserver agreement (*p* < 0.05).

**Results:**

Initial search yielded 603 videos with 13 meeting the inclusion criteria. The injuries were sustained performing a sporting activity (*n* = 9) or whilst dancing (*n* = 4). The injury was predominantly sustained during a non-contact situation (*n* = 10). The most common mechanism was an unbalanced individual with a flexed hip sustaining a valgus force to their flexed knee with the tibia externally rotated.

**Conclusions:**

This study provides some insight into the MoI for a patellar dislocation and the findings may assist in developing injury prevention programmes and rehabilitation protocols as well as guiding future research.

## Introduction

A patella dislocation is a multifactorial phenomenon that can have numerous long-term sequelae [1] Following a single dislocation, a large proportion of patients continue to endure symptoms [2] including pain and recurrent instability, which may result in a restriction in their activites [3]. Our knowledge on the diagnosis and management of patella instability has grown in recent years, but our understanding about the mechanism by which the dislocation occurs remains limited and this is an important element of developing an injury prevention model.

**Fig. 1:**
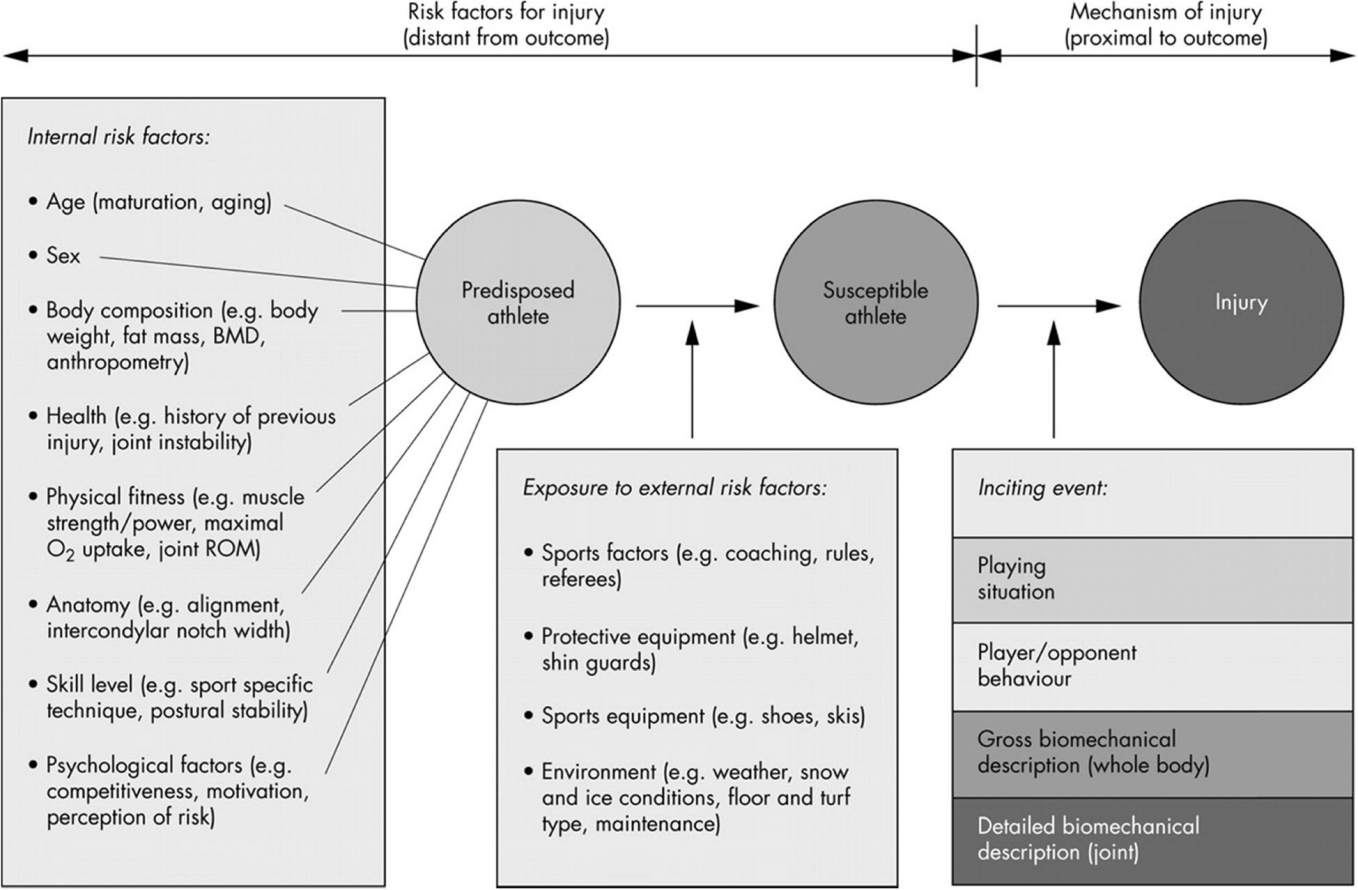
Model developed by Bahr and Krosshaug [5] exploring the various causes of an injury. BMD, bone mineral density; ROM, range of motion

Injury prevention models require both the identification of risk factors and the mechanism of injury (MoI) [4] (Fig. 1). Various methods have been utilised to determine mechanisms including interviews with athletes, clinical studies, laboratory motion analysis and cadaveric studies. Systematic video analysis can be used to gather information on the situation at the time of injury and the individual’s movements. Its use has gained popularity in recent years following work on cervical spine injuries in rugby [6]. This was followed, famously, by the work of Ettlinger [7] in identifying the “phantom foot” mechanism of anterior cruciate ligament (ACL) injury in skiers. They were able to use this understanding to reduce the incidence of ACL injuries by 62% through the development of a video-based training programme.

Systematic video analysis is now a common method of establishing a MoI. In the digital age this has progressed to the utilisation of online videos using websites such as YouTube. This method has been used in assessing the MoI of ankle fractures [8] and elbow dislocations [9].

Whilst various theories on the mechanism of injury of a patella dislocation have been proposed, to date these theories have not been reproduced through video analysis. Previous video analysis has shown that theoretical models of MoI can be misleading, as demonstrated by Schrieber et al. in their analysis of the mechanism of elbow dislocations [9].

The aim of this study was to identify the mechanism of injury of patella dislocations using systematic video analysis of publicly available videos on open-access video sharing websites.

## Method

Three largest online video sharing websites (YouTube, Vimeo and DailyMotion) and three popular Internet search engines (Google, Bing and Yahoo) were systematically searched on 1 February 2017 using combinations of the following search terms: ‘kneecap’ or ‘patella’ with ‘dislocation’, ‘dislocating’ or ‘dislocated’. Videos were initially reviewed by the lead author to confirm patella dislocation. Where possible, media reports were used to provide additional confirmation.

Any video in which the quality was not sufficient to allow review, or in which there was any doubt about the diagnosis, was excluded from further analysis. The inclusion and exclusion criteria can be seen in Table 1.

**Table 1:** Inclusion and exclusion criteria

Inclusion criteria	Exclusion criteria
Video of adequate quality that allows for review Patella dislocation that can be confirmed visually or by another reliable source (i.e. media report and/or uploading individual/organisation)	Video of insufficient quality to allow for review Unable to confirm diagnosis of patella dislocation Videos produced for educational purposes that demonstrate a simulated patella dislocation

Videos were assigned an anonymous study number and each video was reviewed independently by three fellowship trained orthopaedic surgeons with an interest in knee surgery and sports injuries. The reviewers received specific training in systematic video analysis.

Each reviewer conducted the systematic video analyses on “full screen” mode on a monitor of their choice. They were advised to do this on the largest monitor possible and permitted to view the footage as many times as required at the speed that they desired. They were required to report on the position of the lower limb at the time of injury and the circumstances in which it occurs (Table 2). The aim was to report a MoI based on the guidance developed by Bahr and Krosshaug [5] and describe the:

1. Vital aspects of the playing situation
  - Sports-specific description
2. Injured individual’s behaviour at the time of injury
  - E.g. Balance, concentration
3. Whole body biomechanical characteristics
  - E.g. Weight distribution, balance
4. Biomechanical characteristics of the lower limb
  - Hip position
  - Knee position
  - Ankle position
  - Foot position

**Table 2:**
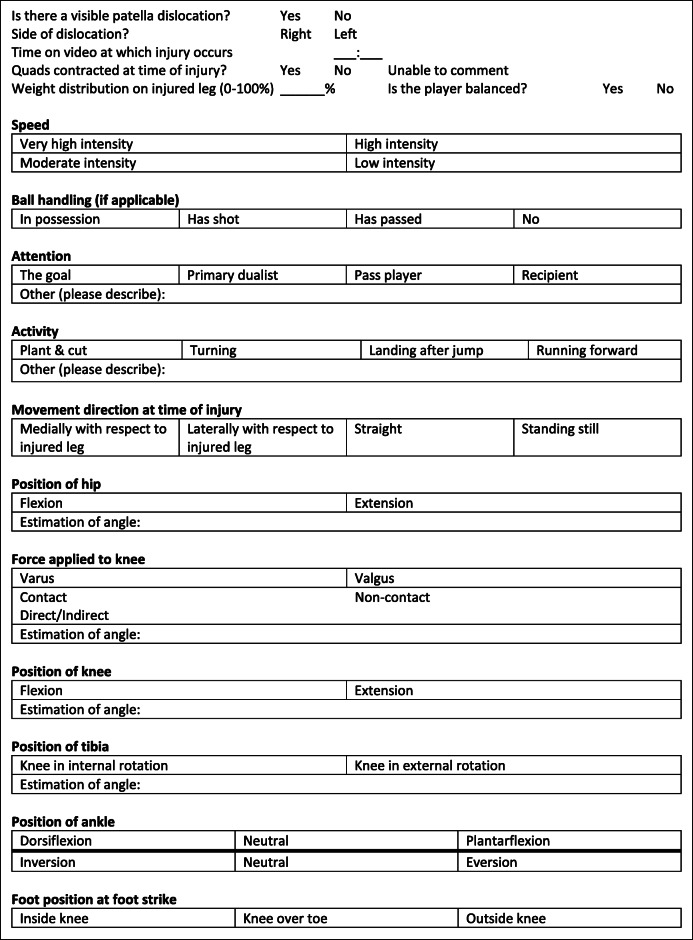
Categories to describe patella dislocation injury and biomechanics through systematic video analysis by reviewers

The reviewers were provided with a guidance tool containing definitions to assist them whilst reviewing. The reviews were conducted independently, and each reviewer was blinded to other reviewers’ results.

Using a similar method to Krosshaug [10], who assessed ACL injuries in basketball players, the resulting analyses were reviewed to build a consensus on the MoI for each video. If the reviews of at least two out of three reviewers were not in agreement on a particular aspect, this was labelled as “no consensus”.

Statistical analysis was conducted using SPSS (IBM, New York, USA). Interobserver agreement was assessed by calculating the interclass correlation coefficient (ICC) with significance set at *p* < 0.05.

## Results

The search yielded a total of 603 videos on YouTube. No additional videos were identified on the other two video sharing websites or search engines. After review of the titles and content, 17 videos were deemed to be suitable for an initial review; 586 videos were excluded because they were either educational videos or on viewing the videos, it was found they did not actually show a patella dislocation occurring. Subsequently, further analysis of these videos meant that four were not suitable for review as it was not possible to confirm the diagnosis in three of them, and the quality of one video was not adequate to allow review.

**Fig. 2:**
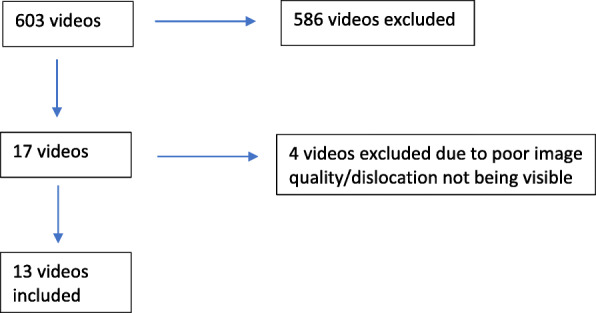
Flow chart demonstrating a list of videos included for review

Therefore, 13 videos were left for review. This has been summarised in Fig. 2 with a list of videos reviewed shown in Table 3. The demographics of the participants in the final 13 videos included in this study are shown in Tables 4 and 5*.*

**Table 3:** Videos demonstrating patella dislocation used for systematic video analysis

Video number	Video title	Address
01	Baron Davis Knee Injury (Patellar Dislocation)- Super Slow Motion	https://www.youtube.com/watch?v=-kRMSYelGTU
02	Patella Dislocation Animation	https://www.youtube.com/watch?v=vpEnUQ3NduQ
03	Patellar Dislocation and MPFL Rupture	https://www.youtube.com/watch?v=gUKRsZN6ZXo
04	Dislocation of right knee cap and tears	https://www.youtube.com/watch?v=dJyhfMyPadw
05	Utah Jazz SG Patrick Christoper dislocated Patella vs. Atlanta Hawks	https://www.youtube.com/watch?v=Vmzm1PU1NJg
06	AFL- Jaymie Graham dislocated kneecap injury (WAFL)	https://www.youtube.com/watch?v=WDhjV0zsoL4
07	Danish Judo Championship (Dislocated kneecap)	https://www.youtube.com/watch?v=GUUY5wbHqqo
08	140 bench knee dislocation	https://www.youtube.com/watch?v=3uIN4PdwgTE
09	Knee dislocation	https://www.youtube.com/watch?v=Oih90HXSjvM
10	Knee cap dislocation!	https://www.youtube.com/watch?v=mhjhZM0fNUg
11	Ouch!!!!.......How to dislocate your knee dancing.	https://www.youtube.com/watch?v=EUYwWAKLPf4
12	Citizen TV’s Willis Raburu injures knee live on air, requests for prayers	https://www.youtube.com/watch?v=yPzmxuvd0yU
13	Grizzlies @ Lakers, 2008 (Bynum knee injury)	https://www.youtube.com/watch?v=sSXxL-UO4fk

**Table 4:** Type and level of activity

Activity		Level	
Basketball	3	Professional	5
Australian rules football	2	Amateur	6
Dance	4	Unknown	2
Judo	1		
Weightlifting	1		
Exercise	1		
Wrestling	1

**Table 5:** Demographics of individual sustaining patella dislocation

Gender		Laterality	
Male	9	Right	5
Female	4	Left	8

### Patella dislocations

There were 12 videos that demonstrated a patella dislocation occurring whilst performing a dynamic action and 1 video that demonstrated a patella dislocation sustained whilst performing a static action (bench press). At the time of injury, it was found that the quadriceps was contracted in 12 out of 13 videos. The reviewers all stated that they were not able to comment on this element in the other video.

### Non-contact patella dislocations

**Fig. 3:**
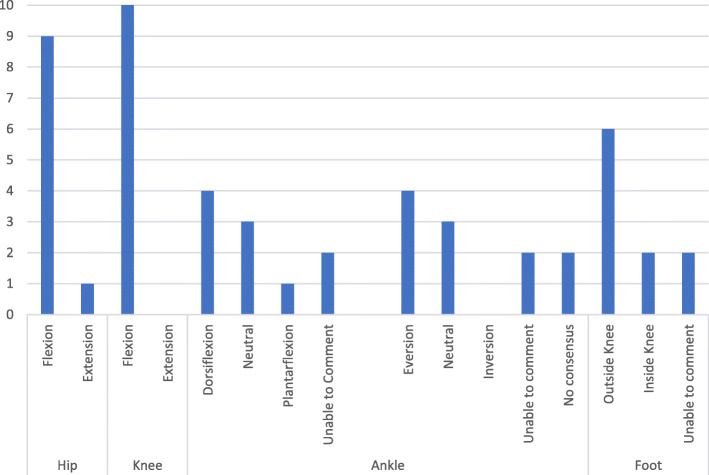
Position of hip, knee, ankle and foot at time of patella dislocation in non-contact dislocations based on consensus of reviewers

Of the 13 patella dislocations, 10 occurred in a non-contact situation, 5 of which were sustained whilst playing a contact sport. Of these 10 individuals, 7 were considered to be off-balance when the injury occurred, 2 were balanced and there was no consensus on balance at the time of dislocation in the remaining individual. Of the 10 non-contact injuries, 5 were sustained whilst the individual was moving at a low intensity (4 were dancing and 1 was doing an exercise activity): only 1 of these individuals was felt to be balanced at the time of dislocation with 3 considered unbalanced, and there was no consensus on the remaining individual. The mean body weight, as estimated by the reviewers, on the injured limb was 70.37%. The ICC for this was 0.410 (*p* > 0.05). The positions of the hip and knee during the dislocation in these 10 cases are shown in Table 6. The position of the ankle varied at the time of injury. The reported positions of the lower limb as determined by the reviewers are shown in Fig. 3*.*

**Table 6:** Mechanism of injury of patella dislocations identified by systematic video analysis

Hip position	Force applied to knee	Knee position	Average knee flexion angle	Tibia position	Number demonstrating MoI
Flexion	Valgus	Flexion	48.96°	External Rotation	8
Flexion	Valgus	Flexion	33.33°	No Consensus	1
Extension	Valgus	Flexion	40.00°	External Rotation	1

### Contact-injury patella dislocations

Two videos demonstrated an individual sustaining a contact injury resulting in a patella dislocation. One injury was sustained during an Australian rules football match and the other whilst wrestling. Reviewers determined that neither individual was balanced and that both injuries were related to a direct valgus blow to a flexed knee. In one situation, the knee was estimated to be flexed approximately 20° with an externally rotated tibia. The other injury was sustained with the knee flexed at approximately 60° with an internally rotated tibia.

### Ball sports

Five videos demonstrated a dislocation happening during ball sports (three basketball videos and two Australian rules football videos). In three of these videos the individual was either carrying the ball or had just received possession of it. In the two remaining videos, they were not deemed to be in possession of the ball, with one individual having just passed the ball and the other involved in a defensive action. They were found to be landing in three videos, running straight in one and performing a plant and cut manoeuvre in the other one. In the videos involving ball sports, the individual was moving at a “very high” (sprinting) or “high” (below sprinting) intensity in four videos and at a low intensity (jogging) in the remaining video.

### Interrater agreement

**Fig. 4:**
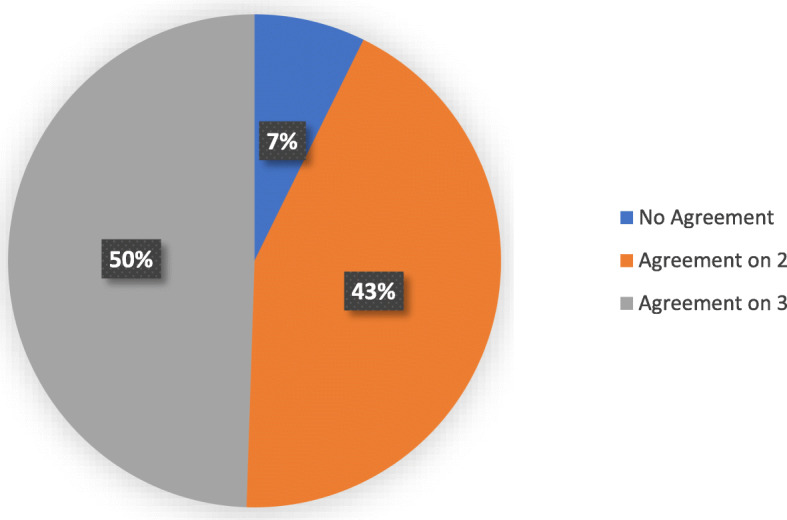
Chart demonstrating level of agreement between reviewers

There was excellent agreement between reviewers with respect to identifying the time at which the injury occurred. All times were within 1 s of each other. Statistical analysis using the ICC gave a value of 1.00 (*p* < 0.05). For the majority of cases the reviewers agreed on two or more parameters. On 50% of occasions they were all in agreement on their analysis of a particular parameter. This can be seen in Fig. 4.

## Discussion

This is the first study using video analysis to determine the mechanism of injury by which a patella dislocation occurs. We have found that the majority of patella dislocations occur in an unbalanced individual during a non-contact situation, where a valgus force is applied to a flexed knee. The quadriceps muscles are contracted at the time of injury, the tibia is externally rotated, and the hip is likely to be flexed. This is in keeping with the theoretical MoI suggested by Hughston [11]. However, we are not able to comment on the degree of hip flexion or tibial external rotation due to the difficulty in assessing these elements from one camera angle. There was also a lack of consensus on the position of the ankle. This may be due to the videos used for analysis not allowing an accurate assessment of the ankle.

Other potential theories have been suggested for the mechanism of injury. Maletius [12] describes it happening in a deeper flexion position in a patient with trochlea dysplasia. Cash and Hughston [13], on the other hand, report that a lateral patellar dislocation may occur as result of a direct blow to either the knee, producing a valgus stress, or the medial edge of the patella. Nikku [14] interviewed 126 people who had suffered a patella dislocation and identified two alternative mechanisms of injury; that of a flexed knee moving into extension whilst accelerating, and a flexed knee moving into deeper flexion whilst decelerating.

A common feature noted in three of the five ball sports videos was the dislocation occurring whilst landing. Biomechanical analysis in predominantly cadaveric studies of the patellofemoral joint have shown that there is a laterally directed force acting upon the patella [15] in normal motion, which has been termed the “law of valgus” [16]. Alterations in the position of the lower limb can influence the force acting upon the patella and result in a dislocation. Single-leg landing has been shown to undergo the same joint reaction force (JRF) on a single knee as double-leg landing where the JRF would be distributed across both knees. Furthermore, upon landing, the hip simultaneously goes into adduction and increased valgus [17]. This, one suspects, would increase the lateral vector force upon the patella and contribute to a greater likelihood of lateral dislocation when landing.

In our series, the speed of the individual’s movement at the time of dislocation varied, as the dislocation occurred at low and high speeds. This variation may represent sampling bias, but it also demonstrates that high speed is not a required risk factor for a patella dislocation to occur.

The majority of videos in this study were of individuals who were participating in a sporting activity of some sort, which is in keeping with the findings of other studies [1, 18]. There may be an element of bias associated with this, however, as those are the types of activities that are likely to be filmed. As with this study, Mitchell [19] in his cross-sectional epidemiological study of high-school athletes in the USA found that the most common reason for a patella dislocation was a non-contact injury. However, they also showed that overall, contact patella dislocations were more common amongst male athletes, whilst female athletes sustained more non-contact patella dislocations. There was an insufficient number of contact injuries in our study to be able to propose or confirm such a trend.

One of the limitations of this study is that we are unable to confirm the diagnosis for all of the videos included in this study. In the case of the professional athletes, there were media reports from the time of the injury that we could access. For other videos, there was a reliance upon the synopsis of the video as the source of the information. On occasion, it was the individual themselves, a family member or, in one case, the individual’s employer who had uploaded the video. Furthermore, we were also unaware of the past medical history of the patient, any risk factors for patella dislocation or whether they had experienced a patella dislocation previously. We know from various biomechanical and kinematic studies that these factors can alter patellar tracking and may, as a result, alter the vulnerable position for them [20]. This, of course, is also true if they have had any other injuries to the knee, have hypermobility or have undergone previous surgery to address patellar problems or any other abnormal pathologic knee conditions. Researchers conducting a similar previous study assessing the mechanism of injury for an ankle fracture did attempt to make contact with patients, but they only achieved a response rate of 6% [8].

A further limitation relates to the quality of the video and the viewing angles available. This was highly variable and very much dependent upon the level of sport. Professional games often have multiple angles available for analysis, whereas those shot by amateur videographers were limited to one angle with low resolution and often unsteady images. This affected the reviewers’ ability to comment on the angle of the hip and position of the tibia at the time of dislocation. It may have also influenced the reviewers’ ability to comment on other elements of the injury mechanism.

Our subgroup analysis of patella dislocations in ball sports has shown that dislocations can occur in a variety of situations, including being in possession of the ball or not in possession, as well as when landing. However, we have not been able to develop a sports-specific injury model owing to the fact that these injuries occurred during different activities. This, also, does not allow us to develop a description of the inciting event or the circumstance leading up to the injury. Ideally such a study would be conducted with a larger sample size, but this may be difficult to achieve. A patella dislocation represents a small proportion of all knee injuries and capturing it can be difficult, especially as many occur in recreational activities. Whilst a limitation of our study is sample size, such small studies are still able to provide valuable information as shown by Longo et al [20]. Their study of four elite-level rugby players sustaining a shoulder dislocation provided preliminary evidence on the mechanism of this injury in rugby.

The use of online video sharing websites presents an interesting opportunity for in vivo analysis of injuries. One area of concern lies in the rights to the uploaded videos. All the videos from this study were obtained from YouTube, which is currently the biggest video sharing website. In their terms of service (YouTube [21]), it states that once content is uploaded, they cannot guarantee any confidentiality and that users of the service are free to use the content as permitted by the functionality of the site [21]. Furthermore, individuals in another similar study commented that their purpose for uploading their video was to allow others to be able to share their experience [8].

## Conclusion

Whilst our case series is small, we believe that we have been able to provide further insight into the circumstances in which a patella dislocation may occur. A patella dislocation is predominantly a non-contact injury. The most common mechanism of a patella dislocation in our series was that of an unbalanced individual with a flexed hip, sustaining a valgus force to their flexed knee with the tibia externally rotated. Our results provide some preliminary evidence that dislocations can occur across a wide variety of activities. Given the mechanism of injury we have identified, there may be a role in incorporating balance and landing exercises designed to prevent a valgus collapse of the knee into rehabilitation regimes and injury prevention programmes. However further research is needed to investigate this further and use the findings to help in the development of such regimes and programmes.

## Data Availability

All relevant data & materials are included in the article.

## Abbreviations

ACL: Anterior cruciate ligament
ICC: Interclass correlation coefficient
JRF: Joint reaction force
MoI: Mechanism of injury
